# Comparison of antimicrobial prescription patterns in calves in Switzerland before and after the launch of online guidelines for prudent antimicrobial use

**DOI:** 10.1186/s12917-020-02704-w

**Published:** 2021-01-05

**Authors:** Alina Hubbuch, Ruth Peter, Barbara Willi, Sonja Hartnack, Cedric Müntener, Hanspeter Naegeli, Christian Gerspach

**Affiliations:** 1 Institute of Veterinary Pharmacology and Toxicology, Vetsuisse Faculty, University of Zurich, Winterthurerstrasse 260, CH-8057 Zurich, Switzerland; 2 Clinic for Small Animal Internal Medicine, Vetsuisse Faculty, University of Zurich, Winterthurerstrasse 260, CH-8057 Zurich, Switzerland; 3 Section of Epidemiology, Vetsuisse Faculty, University of Zurich, Winterthurerstrasse 270, CH-8057 Zurich, Switzerland; 4 Clinic for Ruminants, Department for Farm Animals, Vetsuisse Faculty, University of Zurich, Winterthurerstrasse 260, CH-8057 Zurich, Switzerland

**Keywords:** Food-producing animals, Cattle, Prescription patterns, Prudent use guidelines, Crowding disease, Calf scours

## Abstract

**Background:**

The increasing threat of bacterial resistance promotes the need for antibiotic stewardship programs to foster responsible antimicrobial use. Therefore, guidelines for prudent use supported by an online stewardship tool (AntibioticScout.ch) were introduced in Switzerland in December 2016. They recommend (with decreasing preference) a first, second or third line antimicrobial for treatment. The objective of this study was to evaluate antimicrobial prescriptions for Swiss calves before (2016) and after (2018) the launch of these guidelines. Cases of calves with pneumonia, diarrhea and otitis from a university hospital and eight private practices in Switzerland were included. Data on anamnesis, clinical findings, diagnostic work-up and treatment were collected. Type and percentages [95% confidence interval] of antimicrobial prescriptions were compared between 2016 and 2018.

**Results:**

Of the total number of calves, 88.2% [85.4–90.6] in 2016 (*n* = 625) and 88.4% [85.7–90.7] in 2018 (*n* = 655) were treated with antibiotics. The use of highest priority critically important antimicrobials (HPCIAs) decreased from 52.7% [48.6–56.9] in 2016 to 38.0% [34.2–41.9] in 2018; this decrease was found at the university hospital and in private practice and in cases with pneumonia and diarrhea. Particularly the use of fluoroquinolones decreased (2016: 43.1% [39.2–47.2]; 2018: 31.1% [27.6–34.8]). Overall, the number of first line treatments increased from 12.8% [10.4–15.6] in 2016 to 20.2% [17.3–23.4] in 2018. In cases of pneumonia, first line treatments increased (2016: 15.3% [11.6–19.9]; 2018: 26.5% [21.8–31.9]) and third line treatments decreased (2016: 43.5% [38.0–49.3]; 2018: 27.9% [23.1–33.3]); this was seen at the university hospital, whereas in private practice only a decrease of third line treatments was observed. In cases of diarrhea, more second line at the expense of unlisted antimicrobials were prescribed at the university hospital in 2018. Antimicrobial treatment of calves with otitis did not change from 2016 to 2018.

**Conclusions:**

After the introduction of AntibioticScout.ch, more prudent use was observed in the treatment of calves with pneumonia and diarrhea as less HPCIAs, particularly fluoroquinolones, and more first line antimicrobials were prescribed. However, the overall frequency of antimicrobial treatment did not change and the use of HPCIAs was still common in 2018, especially in private practices. Therefore, further antimicrobial stewardship activities are necessary.

**Supplementary Information:**

The online version contains supplementary material available at 10.1186/s12917-020-02704-w.

## Background

Worldwide, large amounts of antimicrobial agents are used in animals, which foster the development of various antimicrobial resistances through a selection pressure on bacteria [1–3]. In 2016, 7′860 tons of veterinary antimicrobial agents were sold in 30 European countries, while in Switzerland, around 37.5 tons were sold for food-producing animals [4, 5]. Antibiotic-resistant strains, which are frequently detected in calves in Switzerland, might be a consequence. In the period from 2010 to 2017, an increase in methicillin-resistant *Staphylococcus aureus* was measured in slaughter calves and high resistance rates against tetracycline are common among respiratory tract pathogens [6–9]. Management practices like group treatments, external calf purchase, crowding and feeding of milk by-products were found to be risk factors for antimicrobial resistances in Swiss calves [10–12]. The World Health Organization (WHO) defined Highest Priority Critically Important Antimicrobials (HPCIAs), which should be used very prudently in animals to preserve their efficacy for human medicine [13]. Nevertheless, they are commonly prescribed in food-producing animals, especially for the treatment of calves [12, 14–17]. In a Swiss study, fluoroquinolones were used in 65% and 3rd or 4th generation cephalosporins in almost 9% of single animal treatments in calves on fattening farms [18].

Antimicrobial stewardship measures to optimize and reduce antimicrobial use are important [19]. They can include voluntary approaches like improved education, financial regulations, restrictions, preventive actions and alternatives to antibiotics [20, 21]. Various actions have already been implemented. Accordingly, antibiotic sales from 2008 to 2016 decreased by 45% in Switzerland, which is mainly due to decreasing sales of premixes [4]. One method to further improve antibiotic prescriptions are antimicrobial use guidelines. In human medicine, there are many examples on how such guidelines can reduce antimicrobial prescriptions and how compliance with the guidelines can be increased [22–25]. Meanwhile, in farm animal medicine, several prudent use guidelines have been issued as well [26–31]. However, the impact of the guidelines has only been investigated in few studies. In Germany, the implementation of compulsory prudent use guidelines led to a marked reduction of antibiotic consumption in pig production [32]. Additionally, a meta-analysis revealed that interventions restricting antibiotic use in food-producing animals are associated with reduced antimicrobial resistance and suggests that these measures also led to a reduction in antimicrobial resistance in humans, especially in those with exposure to food-producing animals [33].

In December 2016, national guidelines for prudent antimicrobial use were introduced in Switzerland and disseminated through an online decision support tool known as AntibioticScout.ch [34, 35], which include recommendations for the most common indications for antimicrobials in different animal species. These guidelines were established by experts of the Vetsuisse Faculty Bern and Zurich in collaboration with the Swiss Veterinary Society and under the coordination of the Federal Food Safety and Veterinary Office. They represent the first national antimicrobial use guidelines in Switzerland and were generated on the basis of the latest scientific evidence, pharmacological principles, resistance reports and the categorization of critical antibiotics by the WHO [36, 37]. The guidelines recommend a first, second and sometimes third line antimicrobial if antibiotic treatment is justified. The preference should always be given to the first line antimicrobial. If the treatment is not successful, second or third line antimicrobials can be considered. Before a change of antimicrobial treatment, diagnosis and previous therapy (dose, route of application, interval and duration) should be reconsidered, particularly if a third line antimicrobial is chosen. Third line antimicrobials include HPCIAs; their use is highly restricted and only justified after pathogen identification and antimicrobial sensitivity testing.

The aim of this study is to investigate antimicrobial prescription patterns on the example of common diseases in calves (diarrhea, pneumonia and otitis) in a university hospital and eight private practices in Switzerland in 2016 and 2018. Next, the level of compliance with the guidelines is evaluated and prescriptions before (2016) and after (2018) the launch of AntibioticScout.ch are compared.

## Results

### Case characteristics

A total of 625 cases in 2016 were compared to 655 cases in 2018. Details of case characteristics, diagnostic work-up and clinical findings in calves with pneumonia, diarrhea and otitis are displayed in Table 1. No differences were found regarding the sex or pretreatment except that, in the cases with diarrhea, more female and pretreated calves were presented in 2018 than in 2016 (Table 1). The age of the calves was not significantly different between 2016, with a range of 1–20 weeks, and 2018, with a range of 1–24 weeks. In addition, there was no difference in the proportion of calves with pneumonia, where the diagnosis was based on a culture of the tracheobronchial secretion, between 2016 and 2018 (Table 1). This was also evident when cases presented to the university hospital (2016: 5.7% [1.6–14.0]; 2018: 2.9% [0.3–9.9]) and to the private practices (2016: 1.3% [0.3–3.9]; 2018: 0.0% [0.0–1.6]) were considered separately. Overall, the proportion of fecal examination in diarrheic calves was not different between the 2 years (Table 1). However, more tests were performed in the university hospital (2016: 55.6% [43.4–67.3]; 2018: 58.3% [46.1–69.8]) than in private practice (2016: 21.9% [16.6–27.9]; 2018: 19.2% [14.3–25.0]).

**Table 1:** Comparison of case characteristics, diagnostic work-up and clinical findings between 2016 and 2018

Parameter		Pneumonia		Diarrhea		Otitis	
		2016 *n* = 294	2018 *n* = 294	2016 *n* = 296	2018 *n* = 296	2016 *n* = 35	2018 *n* = 65
		% [CI^a^]	% [CI^a^]	% [CI^a^]	% [CI^a^]	% [CI^a^]	% [CI^a^]
Sex	Female^b^	9.2 [6.1–13.1]	12.9 [9.3–17.3]	**10.5 [7.2–14.5]**	**18.9 [14.6–23.9]**	NK^c^	4.6 [1.0–12.9]
	Male^b^	3.7 [1.9–6.6]	4.4 [2.4–7.4]	6.1 [3.6–9.4]	7.1 [4.4–10.6]	NK^c^	4.6 [1.0–12.9]
Pretreatment	Yes^b^	16.3 [12.3–21.1]	20.7 [16.3–25.8]	**6.4 [3.9–9.8]**	**14.2 [10.4–18.7]**	17.1 [6.6–33.6]	30.8 [19.9–43.4]
Diagnostic test^d^	Yes^b^	2.4 [1.0–4.8]	0.7 [0.1–2.4]	30.1 [24.9–35.6]	28.7 [23.6–34.2]	NA^e^	NA^e^
Lethargy	Yes^b^	27.6 [22.5–33.0]	23.5 [18.7–28.7]	34.5 [29.1–40.2]	37.2 [31.6–42.9]	8.6 [1.8–23.1]	13.8 [6.5–24.7]
	No^b^	5.4 [3.1–8.7]	10.9 [7.6–15.0]	9.5 [6.4–13.4]	13.9 [10.1–18.3]	5.7 [0.7–19.2]	6.2 [1.7–15.0]
Inappetence	Yes^b^	17.7 [13.5–22.5]	16.0 [12.0–20.7]	27.4 [22.4–32.8]	23.3 [18.6–28.6]	2.9 [0.1–14.9]	3.1 [0.4–10.7]
	No^b^	5.8 [3.4–9.1]	9.2 [6.1–13.1]	13.9 [10.1–18.3]	12.2 [8.7–16.4]	5.7 [0.7–19.2]	4.6 [1.0–12.9]
Fever (≥39.5 °C)	Yes^b^	39.8 [34.2–45.6]	41.2 [35.5–47.0]	10.1 [6.9–14.2]	13.9 [10.1–18.3]	28.6 [14.6–46.3]	33.8 [22.6–46.6]
	No^b^	13.9 [10.2–18.4]	21.1 [16.6–26.2]	37.8 [32.3–43.6]	42.6 [36.9–48.4]	17.1 [6.6–33.6]	13.8 [6.5–24.7]
Abnormal lung sound^f^	Yes^b^	48.0 [42.1–53.8]	50.3 [44.5–56.2]	NA^e^	NA^e^	NA^e^	NA^e^
Scleral injection	Yes^b^	NA^e^	NA^e^	8.8 [5.8–12.6]	10.1 [6.9–14.2]	NA^e^	NA^e^
	No^b^	NA^e^	NA^e^	**4.1 [2.1–7.0]**	**12.5 [9.0–16.8]**	NA^e^	NA^e^
Signs of sepsis^g^	Yes^b^	NA^e^	NA^e^	1.7 [0.6–3.9]	2.0 [0.7–4.4]	NA^e^	NA^e^
	No^b^	NA^e^	NA^e^	4.7 [2.6–7.8]	7.1 [4.4–10.6]	NA^e^	NA^e^
Bloody diarrhea	Yes^b^	NA^e^	NA^e^	11.1 [7.8–15.3]	12.2 [8.7–16.4]	NA^e^	NA^e^

Non-overlapping CIs are written in bold numbers; ^a^CI, 95% confidence interval; ^b^Values not listed were unknown, therefore percentages do not add up to 100%; ^c^NK, not known; ^d^Culture of bronchoalveolar lavage for pneumonia cases/ Fecal examination for diarrhea cases; ^e^NA, not applicable; ^f^Including increased vesicular sounds; ^g^Left shift with toxic neutrophils in hematology

There were no differences regarding the clinical findings between 2016 and 2018, except that scleral injection was more frequently described as absent in 2018. In both years, fever was more often observed in cases with pneumonia than in cases with diarrhea (Table 1). In cases with pneumonia, concurrent diarrhea was common (2016: 6.1% [3.7–9.5]; 2018: 11.6% [8.1–15.8]) and in cases with diarrhea concurrent pneumonia was often listed as an additional diagnosis (2016: 9.8% [6.7–13.8]; 2018: 14.9% [11.0–19.4]).

### Overall comparison of antibiotic prescriptions

Overall, the proportion of cases treated with antibiotics did not change between 2016 (88.2% [85.4–90.6]) and 2018 (88.4% [85.7–90.7]). This was also found when the university hospital (2016: 90.1% [84.0–94.5]; 2018: 90.8% [84.9–95.0]) and the private practices (2016: 87.6% [84.3–90.4]: 2018: 87.7% [84.6–90.4]) were analyzed separately.

**Fig. 1:**
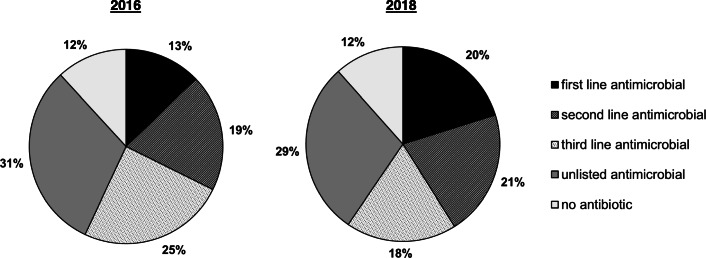
Proportions of prescribed first, second, third line or unlisted antimicrobials and non-antibiotic treatments. 2016: *n* = 625; 2018: *n* = 655

More first line antimicrobials were prescribed in 2018 (20.2% [17.3–23.4]) than in 2016 (12.8% [10.4–15.6]), while for third line antimicrobials, only a tendency towards a reduction was observed (2016: 24.6% [21.4–28.2]; 2018: 18.5% [15.7–21.6]). For second line antimicrobials (2016: 19.5% [16.6–22.8]; 2018: 20.9% [18.0–24.2]) and unlisted antimicrobials (2016: 31.2% [27.7–34.9]; 2018: 28.9% [25.5–32.4]) no changes were observed (Fig. 1).

In the university hospital, more first line (2016: 30.3% [23.3–38.3]; 2018: 51.4% [43.3–59.5]) and second line antimicrobials (2016: 8.5% [4.9–14.2]; 2018: 21.8% [15.8–29.3]) were prescribed in 2018, while proportions of treatments with third line antimicrobials (2016: 20.4% [14.6–27.8]; 2018: 5.6% [2.9–10.7]) and unlisted antimicrobials (2016: 31.0% [24.0–39.0]; 2018: 12.0% [7.6–18.3]) decreased. On the other hand, no changes were observed in private practice with similar proportions of first line (2016: 7.7% [5.6–10.4]; 2018: 11.5% [9.0–14.6]), second line (2016: 22.8% [19.3–26.7]; 2018: 20.7% [17.4–24.4]) and third line antimicrobials (2016: 25.9% [22.2–30.0]; 2018: 22.0% [18.7–25.8]) in both observation periods and similar use of unlisted antimicrobials (2016: 31.3% [27.3–35.5]; 2018: 33.5% [29.6–37.7]). Combination therapies were more often applied in 2018 in private practice (2016: 4.8% [3.0–7.1]: 2018: 9.6% [7.2–12.4]), but not in the university hospital (2016: 4.2% [1.6–9.0]; 2018: 0.7% [0.0–3.9]).

**Fig. 2:**
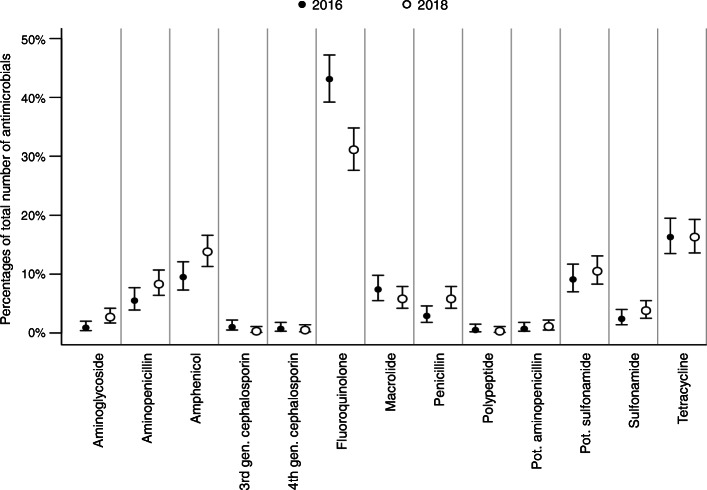
Proportions of prescribed antimicrobial classes in 2016 and 2018. Proportions of prescribed antimicrobial classes (dots) per total number of prescribed antimicrobials in 2016 (*n* = 582) and 2018 (*n* = 640) and corresponding 95% confidence intervals (lines); gen., generation; pot., potentiated

**Fig. 3:**
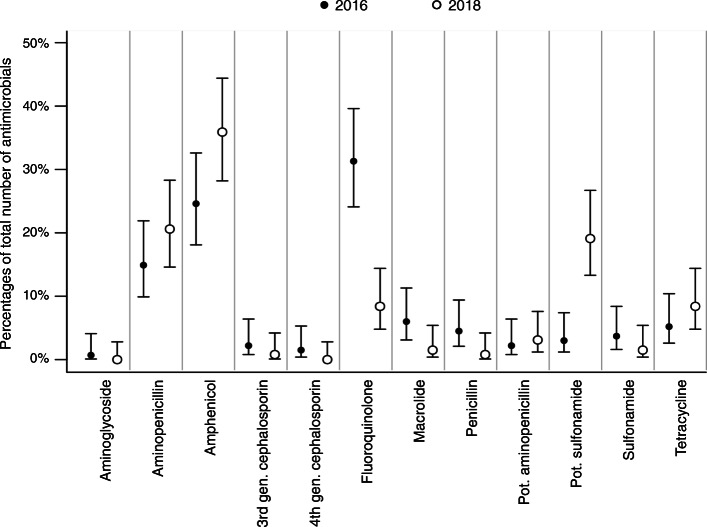
Proportions of prescribed antimicrobial classes in 2016 and 2018; cases presented at the university hospital. Proportions of prescribed antimicrobial classes (dots) per total number of prescribed antimicrobials in 2016 (*n* = 134) and 2018 (*n* = 131) and corresponding 95% confidence intervals (lines); gen., generation; pot., potentiated

**Fig. 4:**
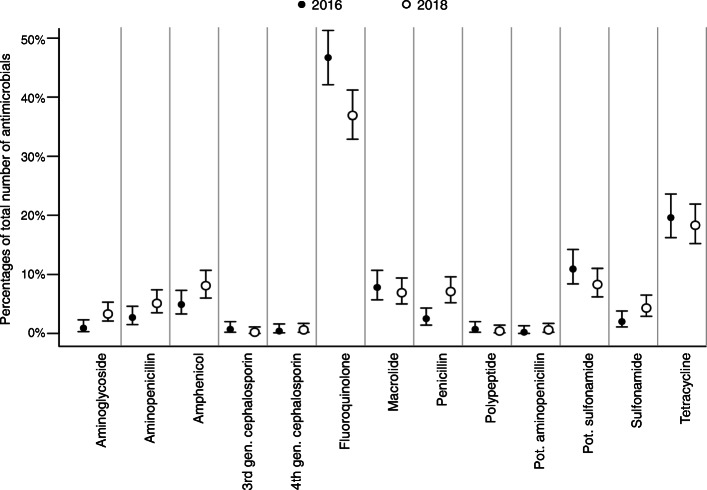
Proportions of prescribed antimicrobial classes in 2016 and 2018; cases presented in private practice. Proportions of prescribed antimicrobial classes (dots) per total number of prescribed antimicrobials in 2016 (*n* = 448) and 2018 (*n* = 509) and corresponding 95% confidence intervals (lines); gen., generation; pot., potentiated

The proportions of prescribed antimicrobial classes overall and separated for the university hospital and private practices are shown in Figs. 2, 3 and 4. Taken together, less fluoroquinolones were prescribed in 2018 (Fig. 2). At the university hospital, there was a marked decrease in the proportion of prescribed fluoroquinolones, while more potentiated sulfonamides were used (Fig. 3). In the private practices, the decrease of fluoroquinolones was accompanied by a more frequent use of penicillins (Fig. 4). The proportion of HPCIAs per total number of prescribed antimicrobials was reduced in all cases (2016: 52.7% [48.6–56.9]; 2018: 38.0% [34.2–41.9], in the university hospital (2016: 41.0% [32.6–49.9]; 2018: 10.7% [6.0–17.3]) and in the private practices (2016: 56.3% [51.5–60.9]; 2018: 45.0% [40.6–49.4]).

In the university hospital, treatment duration did not significantly change with a range of 1–26 days in 2016 and a range of 1–24 days in 2018 (median of 6 days in both years). Treatment duration in private practices could not be assessed, as data on treatment duration was usually not recorded in the case histories. Parenteral injection of the antimicrobials was the most common route of administration (2016: 98.2% [96.7–99.1]; 2018: 97.6% [96.0–98.7]).

### Comparison of antibiotic prescriptions in calves with pneumonia

**Fig. 5:**
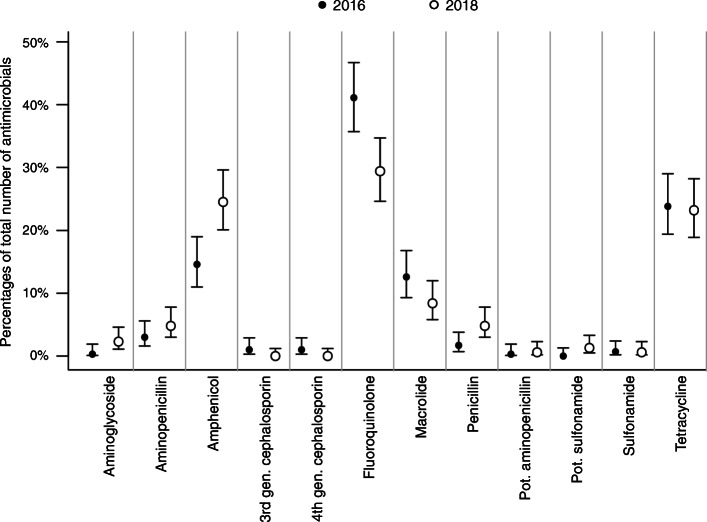
Proportions of prescribed antimicrobial classes in 2016 and 2018; cases with pneumonia. Proportions of prescribed antimicrobial classes (dots) per total number of prescribed antimicrobials in 2016 (*n* = 302) and 2018 (*n* = 310) and corresponding 95% confidence intervals (lines); gen., generation; pot., potentiated

Characteristics of the antimicrobial therapy of calves with pneumonia are displayed in Table 2. More first line antimicrobials were prescribed in 2018 than in 2016. This change was evident in the university hospital but not in private practices, where only a trend was observed. Third line treatments dropped in 2018. The antimicrobial classes used in calves with pneumonia are shown in Fig. 5. A reduction in the use of fluoroquinolones was achieved in 2018, while more amphenicols were used. The proportion of HPCIAs was reduced from 55.6% [49.8–61.3] in 2016 to 37.7% [32.3–43.4] in 2018. This change was evident in the university hospital (2016: 43.5% [31.6–56.0]; 2018: 11.8% [5.2–21.9]) and in the private practices (2016: 59.2% [52.6–65.6]; 2018: 45.0% [38.7–51.5]).

**Table 2:** Comparison of antimicrobial therapy between 2016 and 2018

	Total		University hospital		Private practices	
	2016	2018	2016	2018	2016	2018
**Pneumonia**	*n* = 294	*n* = 294	*n* = 70	*n* = 70	*n* = 224	*n* = 224
	% [CI^a^]	% [CI^a^]	% [CI^a^]	% [CI^a^]	% [CI^a^]	% [CI^a^]
First line antimicrobial	**15.3 [11.6–19.9]**	**26.5 [21.8–31.9]**	**38.6 [28.0–50.3]**	**62.9 [51.1–73.2]**	8.0 [5.1–12.3]	15.2 [11.1–20.5]
Second line antimicrobial	25.2 [20.6–30.4]	30.3 [25.3–35.8]	15.7 [9.0–26.0]	22.9 [14.6–34.0]	28.1 [22.6–34.3]	32.6 [26.8–39.0]
Third line antimicrobial	**43.5 [38.0–49.3]**	**27.9 [23.1–33.3]**	**30.0 [20.5–41.5]**	**7.1 [3.1–15.7]**	**47.8 [41.3–54.3]**	**34.4 [28.5–40.8]**
Unlisted antimicrobial^b^	14.3 [10.7–18.7]	13.6 [10.2–18.0]	12.9 [6.9–22.7]	2.9 [0.8–9.8]	14.7 [10.7–20.0]	17.0 [12.6–22.4]
Non-antibiotic therapy	1.7 [0.7–3.9]	1.7 [0.7–3.9]	2.9 [0.8–9.8]	4.3 [1.5–11.9]	1.3 [0.5–3.9]	0.9 [0.2–3.2]
Combination therapy	4.1 [2.1–7.0]	6.1 [3.7–9.5]	1.4 [0.0–7.7]	0.0 [0.0–5.1]	4.9 [2.5–8.6]	8.0 [4.8–12.4]
**Diarrhea**	*n* = 296	*n* = 296	*n* = 72	*n* = 72	*n* = 224	*n* = 224
	% [CI^a^]	% [CI^a^]	% [CI^a^]	% [CI^a^]	% [CI^a^]	% [CI^a^]
First line antimicrobial	8.1 [5.5–11.8]	13.5 [10.1–17.9]	22.2 [14.2–33.1]	40.3 [29.7–51.8]	3.6 [1.8–6.9]	4.9 [2.8–8.6]
Second line antimicrobial	15.2 [11.6–19.7]	14.5 [11.0–19.0]	**1.4 [0.2–7.5]**	**20.8 [13.1–31.6]**	19.6 [15.0–25.3]	12.5 [8.8–17.5]
Third line antimicrobial	3.7 [2.1–6.5]	2.0 [0.9–4.4]	11.1 [5.7–20.4]	4.2 [1.4–11.5]	1.3 [0.5–3.9]	1.3 [0.5–3.9]
Unlisted antimicrobial^b^	49.7 [44.0–55.3]	46.3 [40.7–52.0]	**48.6 [37.4–59.9]**	**20.8 [13.1–31.6]**	50.0 [43.5–56.5]	54.5 [47.9–60.9]
Non-antibiotic therapy	23.3 [18.9–28.5]	23.6 [19.2–28.8]	16.7 [9.8–26.9]	13.9 [7.7–23.7]	25.4 [20.2–31.5]	26.8 [21.4–32.9]
Combination therapy	4.4 [2.4–7.4]	8.4 [5.5–12.2]	6.9 [2.3–15.5]	1.4 [0.0–7.5]	**3.6 [1.6–6.9]**	**10.7 [7.0–15.5]**
**Otitis**	*n* = 35	*n* = 65				
	% [CI^a^]	% [CI^a^]				
First line antimicrobial	31.4 [18.6–48.0]	21.5 [13.3–33.0]				
Second line antimicrobial	8.6 [3.0–22.4]	7.7 [3.3–16.8]				
Third line antimicrobial	42.9 [28.0–59.1]	50.8 [38.9–62.5]				
Unlisted antimicrobial^b^	17.1 [8.1–32.7]	18.5 [10.9–29.6]				
Non-antibiotic therapy	0.0 [0.0–9.9]	1.5 [0.3–8.2]				
Combination therapy	11.4 [3.2–26.7]	10.8 [4.4–20.9]				

Non-overlapping CIs are written in bold numbers; ^a^CI, 95% confidence interval; ^b^Unlisted antimicrobial, antimicrobial class not listed in the guidelines

### Comparison of antibiotic prescriptions in calves with diarrhea

**Fig. 6:**
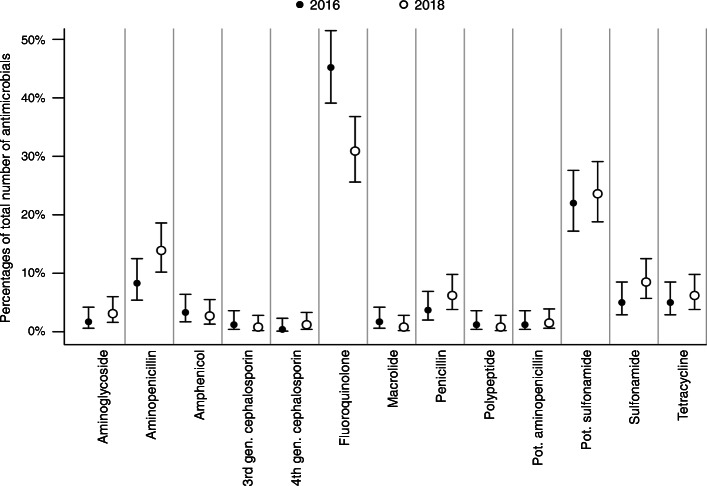
Proportions of prescribed antimicrobial classes in 2016 and 2018; cases with diarrhea. Proportions of prescribed antimicrobial classes (dots) per total number of prescribed antimicrobials in 2016 (*n* = 241) and 2018 (*n* = 259) and corresponding 95% confidence intervals (lines); gen., generation; pot., potentiated

In Table 2, antibiotic therapies of calves with diarrhea are listed. More second line antimicrobials were used in the university hospital in 2018, while less unlisted antimicrobials were used. More combination therapies were applied in the private practices in 2018. Table 3 presents the therapy of diarrhea cases with respect to whether or not antimicrobial use was justified. This analysis did not reveal any changes between the 2 years but, in many cases, the justification for antimicrobial use could not be evaluated because of missing data in the records. The antimicrobial classes prescribed for diarrheic calves are displayed in Fig. 6, showing a reduction in the use of fluoroquinolones in 2018 compared to 2016. There was a reduction in the proportion of prescribed HPCIAs from 49.8% [43.3–56.3] in 2016 to 34.4% [28.6–40.5] in 2018. This reduction was marked in the university hospital (2016: 38.5% [26.7–51.4]; 2018: 9.5% [3.6–19.6], but not evident in private practices (2016: 54.0% [46.3–61.5]; 2018: 42.3% [35.3–49.6]).

**Table 3:** Comparison of justification categories between 2016 and 2018 for calves with diarrhea

	2016 *n* = 296	2018 *n* = 296
	% [CI^a^]	% [CI^a^]
**AMU**^**b**^ **justified**	42.2 [36.5–48.1]	48.3 [42.5–54.2]
a. Antibiotic prescribed	a. 83.2 [75.5–89.3]	a. 86.0 [79.2–91.2]
b. No antibiotic prescribed	b. 16.8 [10.7–24.5]	b. 14.0 [8.8–20.8]
**AMU**^**b**^ **not justified**	5.1 [2.9–8.2]	9.1 [6.1–13.0]
a. Antibiotic prescribed	a. 73.3 [44.9–92.2]	a. 44.4 [25.5–64.7]
b. No antibiotic prescribed	b. 26.7 [7.8–55.1]	b. 55.6 [35.3–74.5]
**Unknown if AMU**^**b**^ **justified**	52.7 [46.8–58.5]	42.6 [36.9–48.4]

^a^CI, 95% confidence interval, ^b^AMU, antimicrobial use

### Comparison of antibiotic prescriptions in calves with otitis

**Fig. 7:**
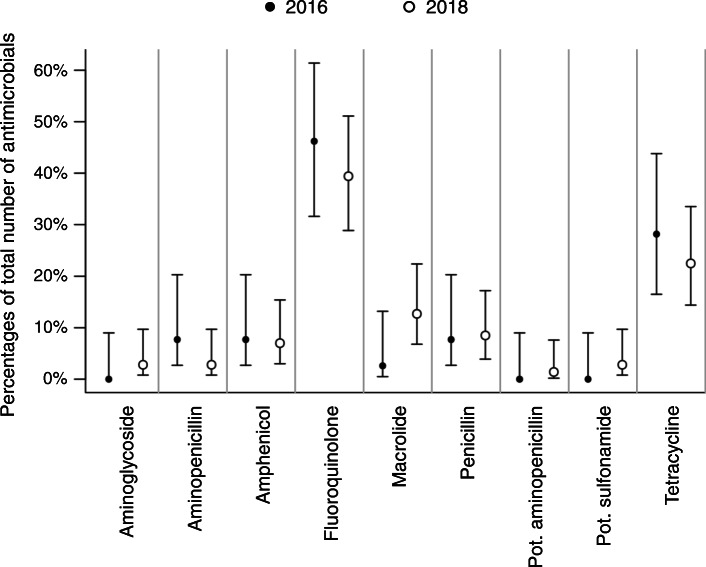
Proportions of prescribed antimicrobial classes in 2016 and 2018; cases with otitis. Proportions of prescribed antimicrobial classes (dots) per total number of prescribed antimicrobials in 2016 (*n* = 39) and 2018 (*n* = 71) and corresponding 95% confidence intervals (lines); pot., potentiated

In cases with otitis an insufficient number of affected calves was found for a sound analysis. Antibiotic therapies and prescribed antimicrobial classes are displayed in Table 2 and Fig. 7, respectively. No differences were found between 2016 and 2018. The proportion of prescribed HPCIAs did not change (2016: 48.7% [32.4–65.2]; 2018: 52.1% [39.9–64.1]).

## Discussion

Our results suggest that after the introduction of prudent use guidelines in Switzerland, less HPCIAs, especially fluoroquinolones, were used and more first line antimicrobials were prescribed to treat calves with pneumonia and diarrhea in the investigated hospital and private practices. This indicates an increase of prudent antimicrobial use and a positive influence of the guidelines on prescription habits of Swiss veterinarians. The decrease in use of preparations classified as HPCIAs for human medicine by the WHO aims to minimize resistance development against these antimicrobial classes. Overall, however, the proportion of antimicrobial prescriptions remained high and almost equal in the two study periods.

The decrease of fluoroquinolone prescriptions from 46.7 to 36.9% in the private practices of the present study and from 31.3 to 8.4% in the investigated university hospital indicate a promising trend towards more prudent use. Previous studies also showed that fluoroquinolones are very commonly used in single animal treatments of calves in Switzerland and other European countries [12, 14, 15, 18]. Two previous Swiss studies reported that fluoroquinolones were prescribed in 65 and 38% of calves during single animal treatments, respectively [12, 18]. In the latter study, the calves were reared under improved welfare conditions and therefore HPCIAs might have been less commonly used. In contrast to other countries in Europe where polymyxins are popular for the treatment of diarrhea in calves, polymyxins were rarely prescribed in our study [14]. There is currently no product containing colistin for oral application in calves available on the Swiss market. One combination product containing amoxicillin and colistin is authorized for parenteral administration but is only rarely used.

The observed increase of first line treatments suggests that the guidelines had a positive impact on prescription habits of Swiss veterinarians in 2018. This was also observed in human and small animal medicine after the implementation of prudent use guidelines [22, 25, 38, 39]. With 20.2%, however, the amount of first line prescriptions in 2018 was still quite low and more first line antimicrobials were prescribed in the university hospital than in private practices (51.4% vs. 11.5%).

Notably, also other changes reveal a more prudent handling of antimicrobials in the university hospital than in private practices. While first and second line antimicrobials increased and treatments with third line or unlisted antimicrobials decreased in the university hospital, no overall changes of antimicrobial treatment were observed in the private practices. Moreover, encouraging changes in the treatment of diarrhea cases were only evident in the university hospital. Finally, the reduction in the use of fluoroquinolones was more marked at the university with a decrease from 31.3 to 8.4% compared to 46.7 to 36.9% in the private practices. It should be mentioned that calves presented to university hospitals are more often pretreated and typically suffer from more severe disease symptoms compared to cases in private practices, which however did not hinder adherence to the guidelines. The higher impact of the guidelines at the investigated university is probably due to the bigger influence of educational actions in this institution and more boarded specialists promoting prudent antimicrobial use. Furthermore, while tracking prescriptions for 1 year should have allowed us to observe unbiased prescription habits of the veterinarians, it is possible that more time is needed for the Swiss guidelines to be fully integrated into the daily routine of the veterinarians. In order to increase the visibility of the guidelines, they are introduced to every veterinary student in Switzerland and in continuing education events. Further activities to foster antimicrobial stewardship include information campaigns for farmers and close monitoring of antimicrobial use.

The most marked changes were observed in the treatment of calves with pneumonia with an increase of first line antimicrobials, especially at the university hospital, and a decrease of third line antimicrobials and HPCIAs both at the university hospital and in private practices. The treatment of pneumonia is quite challenging as antibiotic therapy has to be started as early as possible and resistances in respiratory tract pathogens are quite common in calves in Switzerland [7–9, 40]. Moreover, the frequently involved mycoplasmas are susceptible to only few antibiotics [41]. Therefore, the increased compliance is a positive development but, with 45.0% of treatments with HPCIAs in private practices, further improvements are necessary. Additionally, diagnostic testing was very rare in cases with pneumonia. Treatments based on sensitivity testing and the identification of resistant bacteria would be an important step towards more prudent use and resistance patterns could support evidence-based recommendations for empirical treatments.

Overall, no increase in compliance with the guidelines in cases with diarrhea was observed and combination therapies even increased in private practice. A reduction of the use of HPCIAs was only noted at the university hospital. This is quite surprising as it is controversially discussed if antibiotics are necessary to treat diarrhea at all and the use of HPCIAs is considered obsolete [35, 42, 43]. Concurrent occurrence of pneumonia and diarrhea was common in this study and it was often not distinguishable for which disease the calf was treated. This could be a reason for the frequent prescription of broad-spectrum antibiotics, combination therapies or unlisted antimicrobials. Also, veterinarians could have been tempted to prescribe combination therapies as alternative to the “potent” fluoroquinolones.

In Germany, the introduction of compulsory prudent use guidelines led to a marked reduction of antibiotic consumption in pigs and similar changes were observed in small animal medicine [32, 39, 44]. Furthermore, mortality was not higher after a reduction of antimicrobial use by 46% in a Belgian veterinary practice [45]. Particularly in calves with diarrhea, a decrease of antibiotic treatments is desirable, but was not seen in this study. Responses to questionnaires indicate that farm animal veterinarians are aware of antimicrobial resistance, but believe that the use of antibiotics in food producing animals has no relevant public health impact or that other veterinarians or human medicine mainly contribute to this problem [46–48]. Therefore, some veterinarians may underestimate their potential to combat the development, selection and spread of antimicrobial resistance. Other reasons for not following prudent use guidelines are for example the owners’ request to use a “potent” broad spectrum antibiotic with a short withdrawal period and easy administration. Further, farmers are often reluctant to carry the costs of antibiotic sensitivity testing [49].

For the calves with diarrhea, we tried to assess whether antimicrobial use was justified or not. However, in about 50% of the cases it was not possible to evaluate the necessity for antimicrobial use due to missing information in the records. In the private practices, frequently no information on clinical findings were noted. Criteria whether to prescribe antibiotics or not in diarrheic calves are difficult to define but, due to the risk of bacteremia, it has been recommended to treat all diarrheic calves with signs of systemic illness with antibiotics [43]. In our study, calves with findings indicating a systemic disease such as inappetence, lethargy, fever, engorged scleral blood vessels or signs of sepsis in the hematologic analysis, were judged as cases necessitating antimicrobial use. With these criteria, antimicrobial use was justified in 89.3% (2016) and 84.1% (2018) of the cases where sufficient data were recorded in the case history. It is suggested by the AntibioticScout.ch guidelines to differentiate lethargy due to a possible septicemia from lethargy due to blood acidosis and to assess the mental status after correcting the blood pH with bicarbonate [35]. However, due to the retrospective nature of this study it was not possible to differentiate the causes of lethargy. In a study from Canada, the implementation of a treatment algorithm for diarrheic calves in two farms led to reduced antimicrobial treatment rates without increasing the risk of mortality [50]. In an experimental study, calves showed less days with diarrhea if they were assigned to a targeted therapy group, where diarrhea was only treated with antimicrobials if fever was present [51]. These findings confirm that monitoring the general health status of the calves is a prerequisite to achieve prudent antimicrobial use. The high proportion of cases with justification for antimicrobial use in our study suggests that the criteria were not strict enough as fever was only present in 21.1% (2016) and 24.6% (2018) of the calves in which body temperature was recorded. In calves with pneumonia, instead, it is generally recognized that antimicrobial treatment as early as possible in the course of the disease is necessary in the vast majority of cases [40, 52]. A study that used continuous monitoring of body temperature of the animals found that 25.7% of the calves with fever and a presumptive (not confirmed) diagnosis of pneumonia based on the exclusion of other calf diseases did not require antibiotics after an initial treatment with non-steroidal anti-inflammatory drugs. This study suggests that some cases of pneumonia may resolve without antibiotic treatment [53]. Similar to the treatment recommendations for pneumonia, an early diagnosis and thus a prompt start of treatment is thought to improve the prognosis in cases of otitis [54].

Pneumonia and diarrhea are common diseases in calves and a high frequency of otitis cases was suggested in a Swiss study, where 22.3% of single animal treatments were prescribed for otitis [55]. In the present report, a small number of calves with otitis were available and the cases were very unequally distributed between the different private practices. This is in line with another Swiss study where otitis was only diagnosed in 1.2% of diseased calves [56]. The reason for this discrepancy could be that beef calves were investigated in the first study, whereas the second study focused on dairy calves. Unfortunately, our results indicate that HPCIAs are commonly used to treat otitis. *Mycoplasma bovis* is mainly found in otitis in calves and the chosen antibiotic has to cover this pathogen [41, 54, 57]. Tetracyclines are suggested as first line antimicrobials by the guidelines, but their efficacy may be limited by resistances [7–9].

Digestive disorders and respiratory diseases are considered the main causes of death in Swiss veal calves and antibiotic treatments should be possible in justified cases [58]. Other treatment options like phytotherapeutics and probiotics may be considered [59, 60]. In addition, improved management without transport of animals and crowding as well as prevention by vaccination can reduce the number of diseased calves and, therefore, constitute effective ways to reduce antimicrobial prescriptions [18, 40, 42, 61, 62].

Although only a small sample of veterinary practices was included, our results are reflected by the national antimicrobial sales data. Sales of parenterally administered preparations in Switzerland, which was the main application route for single animal treatment in our study, decreased only by 2.4% between 2016 and 2018, in contrast to the overall sales of antimicrobials that decreased by 15.6% [63]. National sales of HPCIAs including fluoroquinolones were reduced between 2016 and 2018 [63], which was also evident in our study. However, such sales data do not indicate the species or disease for which an antibiotic was used. Our study investigated the prescription patterns before and after the implementation of prudent use guidelines in Switzerland. The true impact of the guidelines could not be unequivocally assessed due to the lack of a control group without access to those guidelines. Since its launch in December 2016, AntibioticScout.ch has been accessed over 275′000 times (as of July 2020), suggesting that the observed shift to more prudent use of antibiotics is largely due to the impact of this new antimicrobial stewardship tool, although we cannot completely rule out that the general awareness on antimicrobial resistance and other educational activities may have contributed to treatment changes. The private practices participated on a voluntary basis and, therefore, were perhaps more inclined to prudent principles than other practices. Moreover, this study revealed that, especially in private practices, the information available in the medical records was often limited. For example, information about treatment duration and justification for antimicrobial use in cases of diarrhea were scarce. The documentation of case histories by farm animal practitioners seems to be poor and electronical records were sometimes used for billing purposes only. Further, follow-up treatments are often allocated to the farmers. Collectively, these prescription habits are not consistent with prudent antimicrobial use as complete medical records are necessary to ensure the compliance with guidelines.

Our data was presented with 95% confidence intervals (CI) and a statistical difference was assumed if CIs were non-overlapping. As a note of caution, this approach does not adjust for multiple comparisons. If restricting our analysis to the overall comparisons of prescribed HPCIAs, fluoroquinolones and first line antimicrobials of significant differences between 2016 and 2018 by Fisher’s exact tests, all *p* values are below 0.001. The significance level of a Bonferroni adjusted *p* value for multiple comparisons would be equal to 0.016.

## Conclusions

In conclusion, we observed a trend towards more prudent antimicrobial use in calves with pneumonia and diarrhea in the investigated university hospital and private practices. These changes were more distinct in the university hospitals. In view of the common poor documentation in medical records, the study underlines the need to better document the data that justify the use and choice of antimicrobials by veterinarians. There is still a large potential to further reduce the prescription of antimicrobials in diseased calves and to handle HPCIAs more prudently, especially in private practices. Therefore, additional antimicrobial stewardship activities to promote prudent use are necessary.

## Methods

Electronic records of the university teaching hospital of the Vetsuisse Faculty Zurich and eight private veterinary practices in Switzerland were studied for calves with pneumonia, diarrhea and otitis. Cases with otitis were only searched in the private practices as this condition is rarely presented to university hospitals. The private practices participated voluntarily and only practices using OblonData® (Amacker&Partner Informatik AG, Zurich, Switzerland) or Diana Suisse® (Diana Software AG, Zurich, Switzerland) were able to participate because of practical reasons. Cases from 2016 were evaluated as base line and cases from 2018 were analyzed as follow-up after introduction of the AntibioticScout.ch tool in December 2016. Records were searched using predefined terms. Calves were defined as cattle under 6 months of age or if the age was unknown but the animal was declared as “calf” in the records, the case was also included. Further, a diagnosis of pneumonia (or if they had a clear history of respiratory signs like coughing or nasal discharge), diarrhea or otitis was necessary for inclusion. Calves were excluded if the animal died or was euthanized before treatment was possible or if the medication was not identifiable. In the private practices, 28 cases per indication and practice were selected. In all but one private practice, less than 28 cases with otitis were found, therefore all cases matching inclusion criteria were included. In the university hospital, all cases found in 2016 were selected for evaluation and the same amount of cases, chosen via random selection, was collected in 2018. Only the initial treatment was evaluated and group treatments were rated as one case.

Data on age, sex, pretreatment, clinical findings, additional diseases, diagnostic work-up (fecal examination and blood hematology for diarrhea, culture of bronchoalveolar lavage for pneumonia) and antibiotic therapy (antimicrobial class, route of administration, treatment duration) were collected. Subsequently, antimicrobial treatment was compared to the consensus guidelines published in AntibioticScout.ch. Antimicrobials were graded as first, second or third line antimicrobial as displayed in Table 4. Antimicrobials not listed in the guidelines were classified as unlisted antimicrobial. Additionally, in cases with diarrhea, clinical findings and hematological results were considered to assess if antimicrobial treatment was justified or not. To facilitate equal judgement and because of limited information in the records, small adjustments of the original guidelines were made (details of the judgement criteria are displayed in Table 5). In cases with pneumonia or otitis, antimicrobial treatment was always considered to be justified.

**Table 4:** Recommendations for antimicrobial choice

Disease	Route of administration	First line antimicrobial	Second line antimicrobial	Third line antimicrobial
**Pneumonia**	parenteral	Florfenicol	Sulfonamide + trimethoprim β-lactam antibiotics^a^ Tetracycline	Fluoroquinolone^b^ 3rd or 4th generation cephalosporin^b^
**Diarrhea**				
caused by enterotoxigenic *Escherichia coli*^c^	oral	Amoxicillin	Sulfonamide + trimethoprim	Neomycin
	parenteral	Amoxicillin	Sulfonamide + trimethoprim	Amoxicillin + clavulanic acid
caused by Coccidia	oral	Sulfonamide		
with suspicion of Salmonellosis	parenteral	Sulfonamide + trimethoprim	Aminoglycoside	Fluoroquinolone^b^
**Otitis**	parenteral	Tetracycline	Florfenicol	Fluoroquinolone^b^ Macrolide^b^

Based on the guidelines for prudent antimicrobial use available on AntibioticScout.ch [34, 35]; ^a^3^rd^ or 4th generation cephalosporin excluded; ^b^highly restricted use, i.e. prescription only justified after pathogen identification and antimicrobial sensitivity testing; ^c^as calves with diarrhea, regardless of etiology, often have coliform bacterial overgrowth of the small intestines [43], cases with unknown cause of diarrhea were judged using the guidelines for diarrhea caused by enterotoxigenic *Escherichia coli*

**Table 5:** Evaluation criteria to assess whether antimicrobial treatment is justified or not in calves with diarrhea

Judgement	Criteria
AMU^a^ is justified if at least **one** of the following criteria is fulfilled^b^:	Lethargy and/or inappetence^c^, fever (≥39.5 °C), scleral injection, signs of sepsis in hematology^d^
Suspicion of Salmonellosis is given if at least **two** of the following criteria are fulfilled^e^:	Fever (≥39.5 °C), hematochezia, signs of sepsis in hematology^d^

Based on the online guidelines for prudent antimicrobial use available on AntibioticScout.ch [34, 35]; ^a^AMU, antimicrobial use; ^b^AMU was defined as not justified if neither lethargy/inappetence nor fever were present; ^c^According to the original guidelines, AMU is only justified in lethargic calves, which don’t improve after correction of the blood acidosis with bicarbonate infusions; ^d^Left shift with toxic neutrophils; ^e^In severe and acute cases, special guideline recommendations can be followed before results of bacteriological culture are available

Highest Priority Critically Important Antimicrobials (HPCIAs) included quinolones, third or higher generation cephalosporins, macrolides, ketolides, glycopeptides and polymyxins as proposed by the WHO [13] (ketolids and glycopeptides have no marketing authorization for veterinary medicine in Switzerland).

IBM SPSS Statistics 23® (IBM, New York, USA) and the software R version 3.6.1 (R Foundation for Statistical Computing, Vienna, Austria [64]) were used for statistical analysis. Interval data (age, treatment duration) were compared with a Mann Whitney U Test. The command binom.test() [64] was used to estimate proportions and Clopper Pearson 95% CIs to compare categorical parameters (case characteristics, diagnostic work-up, clinical findings, justification categories, prescribed HPCIAs and combination therapies). 95% CIs for multinomial proportions were estimated with the command MultinomCI() in the package DescTools [65] for the prescribed antimicrobial classes and to compare first, second, third line antimicrobial treatments, treatments with unlisted antimicrobials and non-antibiotic therapies. With the aim to describe the magnitude of the differences in the investigated parameters, we decided to present 95% CIs, assuming that non-overlapping CIs indicate statistical differences with p smaller as 0.05 [66]. The absolute numbers used to calculate proportions and 95% CIs are available in Additional file 1, Additional file 2 and Additional file 3.

## Supplementary Information

**Additional file 1.** Comparison of case characteristics, diagnostic work-up and clinical findings between 2016 and 2018. Absolute numbers used to calculate proportions and 95% confidence intervals.

**Additional file 2.** Comparison of antimicrobial therapy between 2016 and 2018. Absolute numbers used to calculate proportions and 95% confidence intervals.

**Additional file 3.** Comparison of the justification categories between 2016 and 2018 in calves with diarrhea. Absolute numbers used to calculate proportions and 95% confidence intervals.

## Data Availability

The datasets used and analyzed during the current study are either included in the additional files or available from the corresponding author on reasonable request. The original data were not publically available. Each participating practice signed a written agreement before the data was accessed. Data from owners or origin of the calves were deleted and not available for the study. At the university, owners sign a written agreement, that data can be used for research purposes.

## Abbreviations

CI: Confidence interval
HPCIA: Highest priority critically important antimicrobial
WHO: World Health Organization
AMU: Antimicrobial use
NA: Not applicable
Gen.: Generation
Pot.: Potentiated
