# Supplementary material for: Comparison of antimicrobial prescription patterns in calves in Switzerland before and after the launch of online guidelines for prudent antimicrobial use
Source: BMC Vet Res. 2021 Jan 5;17:2. doi: 10.1186/s12917-020-02704-w (PMC7786965; doi:10.1186/s12917-020-02704-w)
Supplement: Supplementary file 1 — Additional file 1. Comparison of case characteristics, diagnostic work-up and clinical findings between 2016 and 2018. Absolute numbers used to calculate proportions and 95% confidence intervals. [file 12917_2020_2704_MOESM1_ESM.pdf]

Additional file 1: Comparison of case characteristics, diagnostic work-up and clinical findings between 2016 and 2018.

Parameter		Pneumonia		Diarrhea		Otitis	
		2016	2018	2016	2018	2016	2018
		n = 294	n = 294	n = 296	n = 296	n = 35	n = 65
Sex	Female[a]	27	38	31	56	0	3
	Male[a]	11	13	18	21	0	3
Pretreatment	Yes[a]	48	61	19	42	6	20
Diagnostic test[b]	Yes[a]	7	2	89	85	NA[c]	NA[c]
Lethargy	Yes[a]	81	69	102	110	3	9
	No[a]	16	32	28	41	2	4
Inappetence	Yes[a]	52	47	81	69	1	2
	No[a]	17	27	41	36	2	3
Fever (≥39.5°C)	Yes[a]	117	121	30	41	10	22
	No[a]	41	62	112	126	6	9
Abnormal lung sound[d]	Yes[a]	141	148	NA[c]	NA[c]	NA[c]	NA[c]
Scleral injection	Yes[a]	NA[c]	NA[c]	26	30	NA[c]	NA[c]
	No[a]	NA[c]	NA[c]	12	37	NA[c]	NA[c]
Signs of sepsis[e]	Yes[a]	NA[c]	NA[c]	5	6	NA[c]	NA[c]
	No[a]	NA[c]	NA[c]	14	21	NA[c]	NA[c]
Bloody diarrhea	Yes[a]	NA[c]	NA[c]	33	36	NA[c]	NA[c]

The absolute number of cases is indicated for each category; [a]Values not listed were unknown; [b]Culture of bronchoalveolar lavage for pneumonia cases/ Fecal examination for diarrhea cases; [c]NA, not applicable; [d]Including increased vesicular sounds; [e]Left shift with toxic neutrophils in hematology
