# Supplementary material for: Comparison of antimicrobial prescription patterns in calves in Switzerland before and after the launch of online guidelines for prudent antimicrobial use
Source: BMC Vet Res. 2021 Jan 5;17:2. doi: 10.1186/s12917-020-02704-w (PMC7786965; doi:10.1186/s12917-020-02704-w)
Supplement: Supplementary file 2 — Additional file 2. Comparison of antimicrobial therapy between 2016 and 2018. Absolute numbers used to calculate proportions and 95% confidence intervals. [file 12917_2020_2704_MOESM2_ESM.pdf]

Additional file 2: Comparison of antimicrobial therapy between 2016 and 2018.

	Total		University hospital		Private practices	
	2016	2018	2016	2018	2016	2018
**Pneumonia**	n = 294	n = 294	n = 70	n = 70	n = 224	n = 224
First line antimicrobial	45	78	27	44	18	34
Second line antimicrobial	74	89	11	16	63	73
Third line antimicrobial	128	82	21	5	107	77
Unlisted antimicrobial[a]	42	40	9	2	33	38
Non-antibiotic therapy	5	5	2	3	3	2
Combination therapy	12	18	1	0	11	18
**Diarrhea**	n = 296	n = 296	n = 72	n = 72	n = 224	n = 224
First line antimicrobial	24	40	16	29	8	11
Second line antimicrobial	45	43	1	15	44	28
Third line antimicrobial	11	6	8	3	3	3
Unlisted antimicrobial[a]	147	137	35	15	112	122
Non-antibiotic therapy	69	70	12	10	57	60
Combination therapy	13	25	5	1	8	24
**Otitis**	n = 35	n = 65				
First line antimicrobial	11	14				
Second line antimicrobial	3	5				
Third line antimicrobial	15	33				
Unlisted antimicrobial[a]	6	12				
Non-antibiotic therapy	0	1				
Combination therapy	4	7				

The absolute number of cases is indicated for each category; [a]Unlisted antimicrobial, antimicrobial class not listed in the guidelines
