# Supplementary material for: Comparison of antimicrobial prescription patterns in calves in Switzerland before and after the launch of online guidelines for prudent antimicrobial use
Source: BMC Vet Res. 2021 Jan 5;17:2. doi: 10.1186/s12917-020-02704-w (PMC7786965; doi:10.1186/s12917-020-02704-w)
Supplement: Supplementary file 3 — Additional file 3. Comparison of the justification categories between 2016 and 2018 in calves with diarrhea. Absolute numbers used to calculate proportions and 95% confidence intervals. [file 12917_2020_2704_MOESM3_ESM.pdf]

Additional file 3: Comparison of the justification categories between 2016 and 2018 in calves with diarrhea.

	**2016**	**2018**
	n = 296	n = 296
AMU[a] justified	125	143
a. Antibiotic prescribed	a. 104	a. 123
b. No antibiotic prescribed	b. 21	b. 20
AMU[a] not justified	15	27
a. Antibiotic prescribed	a. 11	a. 12
b. No antibiotic prescribed	b. 4	b. 15
Unknown if AMU[a] justified	156	126

The absolute number of cases is indicated for each category; [a]AMU, antimicrobial use
